# Hyperoxia shows duration-dependent effects on the lengths of cell cycle phases in fetal cortical neural stem cells

**DOI:** 10.3389/fcell.2025.1546131

**Published:** 2025-01-28

**Authors:** Jennifer Lanto, Monika Maria Nicole Vehlken, Valeriia Abramenko, Alexander Storch, Franz Markert

**Affiliations:** ^1^ Department of Neurology, University of Rostock, Rostock, Germany; ^2^ German Centre for Neurodegenerative Diseases (DZNE) Rostock/Greifswald, Rostock, Germany

**Keywords:** oxygen, neural stem cells, hypoxia, hyperoxia, physioxia, cortex, cell cycle phases, proliferation

## Abstract

Fetal neural stem cells (NSCs) physiologically reside under low-oxygen conditions (1%–5% of tissue pO_2_), but are often transferred and maintained under atmospheric oxygen levels of 21% pO_2_ (hyperoxia) for *in vitro* investigations. These altered oxygen conditions lead to adaptive changes in NSCs which complicate the interpretation of *in vitro* data. However, the underlying adaption dynamics remain largely enigmatic. Here we investigated short-term hyperoxia effects (5 days in 3% pO_2_ followed by 2 days in 21% pO_2_) in comparison to continuous hyperoxia effects (7 days in 21% pO_2_) and physioxic control (7 days in 3% pO_2_). We utilized cortical NSCs to analyze the cell cycle phases by flow cytometry and cumulative BrdU incorporation assay. NSCs showed a severe reduction of cell proliferation when cultivated under continuous hyperoxia, but no changes after short-term hyperoxia. Subsequent cell cycle analysis as assessed by flow cytometry revealed a clear shift of NSCs from G0/G1-phase towards S- or G2/M-phase after both continuous and short-term hyperoxia. However, while cell cycle length was dramatically reduced by short-term hyperoxia, it was increased during continuous hyperoxia. Taken together, our results demonstrate the beneficial effect of physioxia for expanding NSCs *in vitro* and reveal differential effects of short-term hyperoxia compared to continuous hyperoxia.

## 1 Introduction

Oxygen is a crucial factor during brain development where altered levels of oxygen lead to severe changes of neural stem cell (NSCs) characteristic (Schneider et al., 2012; Lange et al., 2016; Chou et al., 2022). Interestingly, in cortical brain tissue as well as other stem cell niches, NSCs physiologically exist under low levels of oxygen with atmospheric oxygen tensions between 1% and 5% (Dings et al., 1998; Erecinska and Silver, 2001; Lange et al., 2016). It has been shown for cortical NSCs as well as midbrain NSCs that short-term hyperoxygenation over two consecutive days during the mid-neurogenesis phase (E14-E16) *in utero* significantly influences neurogenesis in those regions during brain development demonstrating the pivotal importance of correct oxygen tension within the neurogenic niches (Wagenführ et al., 2015; Wagenfuhr et al., 2016; Markert and Storch, 2022). Indeed, increased oxygen tension in fetal mouse forebrain *in vivo* provokes a marked expansion of a distinct proliferative cell population, basal to the subventricular zone (SVZ) constituting a novel neurogenic cell layer, similar to the outer SVZ, and contributing to corticogenesis. In large contrast, when exposed to prolonged hyperoxia, NSCs show decreased mitosis rate and a reduced overall NSCs amount *in vivo* (Lange et al., 2016; Markert et al., 2020).

In cell culture experiments, the differences between the various durations of hyperoxygenation are closely reflected on the cellular level by initial adaptation of energy metabolism related genes after short-term exposure to hyperoxia over 2 days, while after 13 days exposure to hyperoxia Notch signaling among others was affected (Braunschweig et al., 2022). The parallel decrease of gene hits in metabolic processes in conjunction with an increase in biological regulation and signaling furthermore points to a switch towards signaling processes and stem cell maintenance over time. Mechanistically, cells are able to respond very rapidly to lower oxygen conditions via hypoxia-inducible factor (HIF-1α), and the adaptation process may be driven by a dynamic pattern, as prolonged exposure to 5% oxygen tension over many passages activates subunit HIF-2α more strongly (Forristal et al., 2010). Nevertheless, many *in vitro* investigations are performed under atmospheric oxygen tension (21% pO_2_), a condition which reflects hyperoxia for NSCs. In agreement with their *in vivo* properties, culturing NSCs under hyperoxia leads to reduced proliferation, increased neuro-glial differentiation as well as potentially altered apoptosis (Milosevic et al., 2005; Chen et al., 2007; Francis and Wei, 2010; Dey et al., 2023). Vice versa, when NSCs are cultured under physioxia (3% pO_2_), they show increased proliferation via Wnt signaling and a reduction in their differentiation potential (Zhu et al., 2005; Braunschweig et al., 2015). This severe affection of NSCs by their environment demonstrates the importance of considering oxygen, among other factors, for interpretation of cell culture data. Therefore, we here investigated whether the proliferation potential of cortical NSCs *in vitro* is altered in hyperoxia (21% pO_2_) with respect to physioxia (3% pO_2_) and specifically addressed duration-depend effects of hyperoxia in accordance with *in vivo* data, thereby providing a suitable model to explore molecular mechanisms of oxygen adaptation.

## 2 Material und methods

### 2.1 Cell isolation, seeding and cultivation

All animals were kept in accordance with the German Animal Welfare Act and Directive 2010/63/EU and euthanized in accordance with section 4 of the German Animal Welfare Act. Neural stem cells (NSCs) were isolated from the cortex of C57BL/6J embryos at embryonic day E14. Since the sex of E14 embryos is morphologically indistinguishable, embryos independent of their sex were used for NSC isolation. Tissues were trypsinized for 10 min at 37°C (Trypsin/EDTA 0.05%) and incubated with DNase Ι (0.040 mg/mL) and then minced to obtain a single-cell suspension. The cell suspension was seeded onto PEI-Laminin-coated 6-well cell culture plates (Greiner Bio-One) and 8-well chamber slides (Ibidi) in expansion medium (67% DMEM, 32% F12 + Glutamax, B27, Anti-Anti, EGF, FGF) and cultured for 5 days under physiological conditions (92% N_2_, 5% CO_2_, and 3% O_2_) followed by a 2-day of hyperoxia (5% CO_2_, and 21% O_2_). Controls were cultured under conditions of continuous physioxia (92% N_2_, 5% CO_2_, and 3% O_2_) and continuous hyperoxia (5% CO_2_, and 21% O_2_). To maintain a constant oxygen environment, media for seeding and media changes were pre-equilibrated for 24 h within the respective oxygen condition.

### 2.2 Flow cytometry

After 7 days of cultivation under the respective oxygen conditions, NSCs were dissociated with Accutase. To determine cell density for subsequent staining, cell counting was performed using Countess II. Afterwards, cells were fixed and stained with FxCycle™ PI/RNase Staining Solution (Invitrogen) for 30 min according to manufacturer instructions. Flow cytometry was conducted using the BD FACSCalibur™ cytometer. Data analysis was performed using FlowJo 10.08.1.

### 2.3 Immunocytochemistry

NSCs were fixated by 4% paraformaldehyde after 7 days of cultivation in the respective oxygen condition. After permeabilization by TBST with 0.2% Triton-X 100, NSCs were incubated with the following primary antibodies overnight at 4°C: Anti-Phospho-Histone H3 (RRID:AB_331748, 1:400), and Anti-Cleaved-Caspase-3 (RRID:AB_2341188, 1:400). Subsequently, slides were incubated with corresponding secondary antibodies (Fisher Scientific GmbH, Schwerte, Germany) and cells were covered with Fluoromount-G™ Mounting Medium (Fisher Scientific GmbH).

### 2.4 RNA isolation and qPCR

Total RNA was isolated using the QIAwave DNA/RNA Mini Kit according to the manufacturer instructions. The determined RNA was transcribed into cDNA using the QuantiNova Reverse Transcription Kit. 20 ng of cDNA were used for Real Time Quantitative PCR with Qiagen Rotor-Gene using QuantiNova SYBR Green PCR kit according to manufacturer instructions. Delta-delta-ct method was used for quantitative analysis and Hydroxymethylbilane synthase (*Hmbs*) was used as housekeeping gene. The following cDNA-qPCR primers were used (Eurofins Genomics, Ebersberg, Germany): *Hmbs* (FP: TGA​AAT​CAT​TGC​TAT​GTC​CAC​CAC​G, RP: TCA​GGG​AGT​GAA​CGA​CCA​GG), *Cdk2* (FP: TCA​TGG​ATG​CCT​CTG​CTC​TCA​C, RP: TGA​AGG​ACA​CGG​TGA​GAA​TGG​C, *Cdk4* (FP: TCT​TAG​CCG​AGC​GTA​AGA​TCC​C, RP: GGA​TCT​CGG​GCT​TTG​TAC​ACC), *Cdk6* (FP: GGC​AAA​GAC​CTA​CTT​CTG​AAA​TGC, RP: GGC​AAA​GAC​CTA​CTT​CTG​AAA​TGC), *Ccnd1* (FP: CTT​GAA​GAA​GAG​CCG​CCT​GC, RP: CAG​AAG​CAG​TTC​CAT​TTG​CAG​C), *Ccnd2* (FP: TGA​GCA​CAT​CCT​TCG​CAA​GC, RP: TGG​CAA​ACT​TGA​AGT​CGG​TAG​C), *Ccne1* (FP: CAC​CAC​TGA​GTG​CTC​CAG​AA, RP: CTG​TTG​GCT​GAC​AGT​GGA​GA), *p21Cip1* (FP: GCA​GAT​CCA​CAG​CGA​TAT​CCA, RP: AAC​AGG​TCG​GAC​ATC​ACC​AG).

### 2.5 Cumulative BrdU incorporation assay

After 5 days under the respective oxygen conditions, cells were treated with 10 μM BrdU (5-Bromo-2′-deoxyuridine, SERVA Electrophoresis GmbH). At intervals of 4 h, 6 h, 8 h, 12 h, 16 h, 24 h, 36 h and 48 h NSCs were fixed with 4% paraformaldehyde for 20 min and the reaction was stopped with 4% NH_4_Cl for 10 min. Cells were permeabilized by TBST with 0.2% Triton X-100, DNA was hydrolyzed with 2 M HCl for BrdU staining and non-specific bonds were blocked with a solution of TBST with 2% donkey serum and 1% BSA for 1 h at room temperature. NSCs were incubated with Anti-BrdU (RRID: AB_2536438, 1:200), Anti-Cleaved Caspase-3 (RRID: AB_2341188, 1:800) overnight at 4°C and incubated with corresponding secondary antibodies (Fisher Scientific GmbH). Lastly, Hoechst 33,342 (1:2000) was used to counterstain cell nuclei and slides were covered with Fluoromount-G™ Mounting Medium. By analyzing the BrdU incorporation rate, defined here as the increase of the proportion of BrdU^+^ cells relative to the total cell count (Hoechst^+^ cells) over time, we estimated the total duration of the cell cycle (T_C_), the duration of the S-phase (T_S_), and the proportion of proliferating cells in the population, referred to as the growth fraction (GF). These estimates were obtained using linear regression of the initial increase in BrdU^+^ cells after BrdU exposure, following the method outlined by Nowakowski and colleagues (Nowakowski et al., 1989).

### 2.6 Brightfield and immunofluorescence imaging

NSCs were continuously monitored by standard microscopy and bright-field images were taken using an Axio Imager.A1 (Carl Zeiss, Oberkochen, Germany) with ×10 objective after 7 days of cultivation within the respective oxygen condition. Immunofluorescence images were taken with an Axio Observer.Z1 microscope using a ×20 objective and ZEN blue software with the Tiles and Position module (Carl Zeiss). At least 10 images were taken randomly of each well. Image analyses were conducted using ZEN blue software with analysis module.

### 2.7 Statistical analysis

Data were statistically analyzed in IBM SPSS Statistics 27. If normal distribution and variance equality were met, a one-way ANOVA with Bonferroni adjusted *post-hoc* Student’s *t*-tests were conducted. If these assumptions were not met, Kruskal–Wallis test with Dunn’s *post-hoc* test with Bonferroni correction was applied. Diagrams display mean, standard error of mean (s.e.m.) and individual data points representing results from independent experiments. Graphs were generated using GraphPad Prism9.

## 3 Results

### 3.1 Continuous hyperoxia but not short-term hyperoxia severely reduce NSCs expansion

**FIGURE 1:**
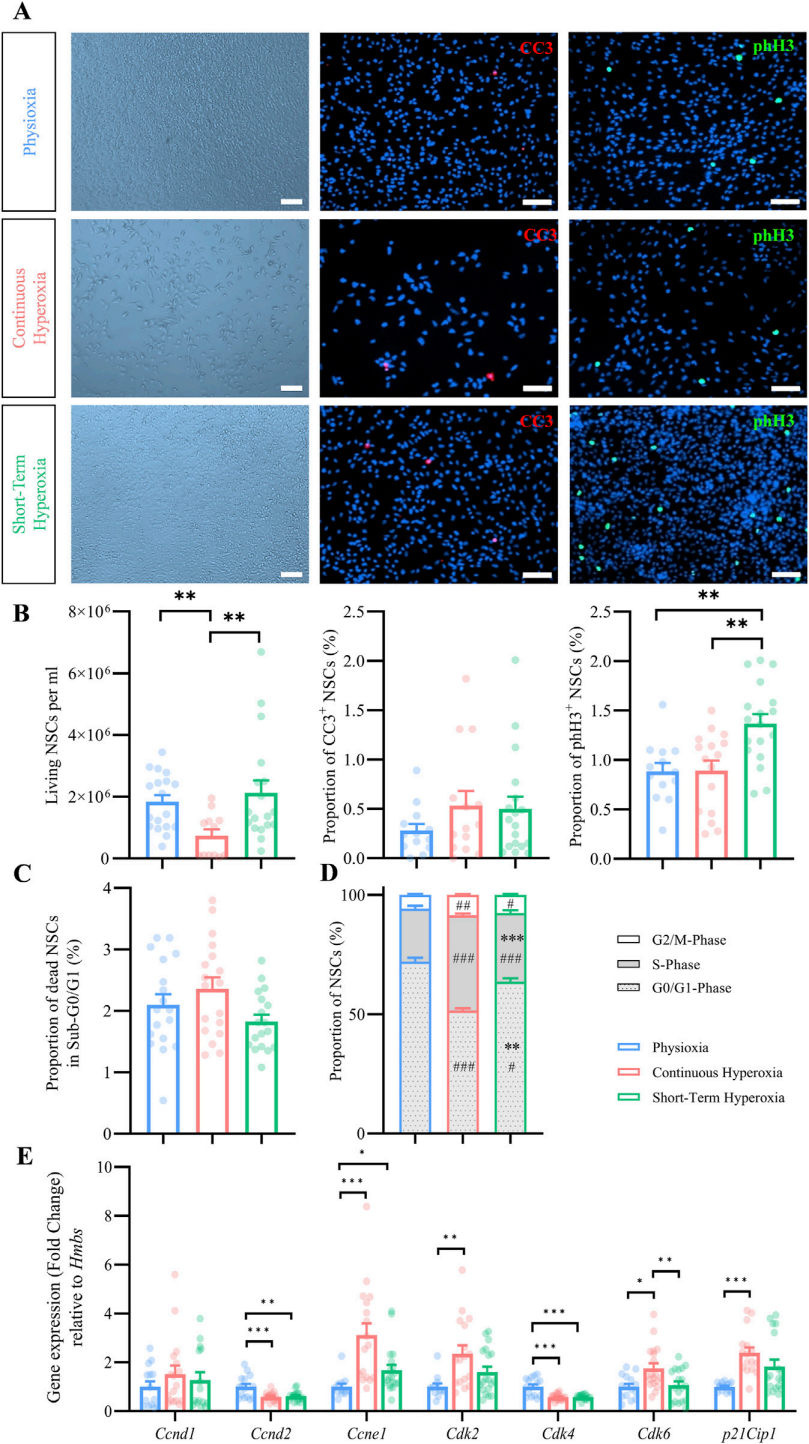
Effects of timed oxygen conditions on NSC expansion, proliferation and death. **(A)** Representative brightfield images of NSCs (left panel) and fluorescence images of CC3^+^ (red, middle panel) and of phH3^+^ (green, right panel) under physiological oxygen conditions (7 days in 3% pO_2_, top), continuous hyperoxia (7 days in 21% pO_2_, mid) and short-term hyperoxia (5 days in 3% pO_2_ followed by 2 days in 21% pO_2_, bottom). Scale bars 50 µm. **(B)** Quantification of living NSCs, stained with trypan blue and analyzed using countess II and of the proportion of CC3^+^ and phH3^+^ NSCs after cultivation in physioxia (blue), continuous hyperoxia (red) or short-term hyperoxia (green). One-way ANOVA with Bonferroni adjusted *post-hoc* test or Kruskal–Wallis with Dunn’s *post-hoc* tests and Bonferroni correction. ***p < 0.001 **p < 0.01 *p < 0.05, independent experiments: n ≥ 13 (physioxia), n ≥ 12 (continuous hyperoxia), n = 18 (short-term hyperoxia). **(C)** Quantification of the proportion of dead NSCs in Sub-G0/G1-phase analyzed by PI flow cytometry after cultivation in physioxia (blue), continuous hyperoxia (red) or short-term hyperoxia (green). Kruskal–Wallis with Dunn’s *post-hoc* tests and Bonferroni correction. ***p < 0.001, independent experiments: n = 18 (physioxia), n = 18 (continuous hyperoxia), n = 18 (short-term hyperoxia). **(D)** Quantification of the proportion of NSCs in G0/G1- (grey dotted), S- (grey), and G2/M-phase (white) analyzed by PI flow cytometry after cultivation in physioxia (blue), continuous hyperoxia (red) and short-term hyperoxia (green). One-way ANOVA with Bonferroni adjusted *post-hoc* tests. # significant compared to physioxia, #p < 0.05, ##p < 0.01, ###p < 0.001; * significant compared to continuous hyperoxia, *p < 0.05, **p < 0.01, ***p < 0.001, independent experiments: n = 18 (physioxia), n = 17 (continuous hyperoxia), n = 18 (short-term hyperoxia). **(E)** Gene expression of cell cycle relevant genes after cultivation in physioxia (blue), continuous hyperoxia (red) or short-term hyperoxia (green), normalized to *Hmbs* and to an internal calibrator of the physioxia data. One-way ANOVA with Bonferroni adjusted *post-hoc* test and Kruskal–Wallis with Dunn’s *post-hoc* tests and Bonferroni correction. ***p < 0.001 **p < 0.01, independent experiments: n ≥ 12 (physioxia), n ≥ 15 (continuous hyperoxia), n ≥ 12 (short-term hyperoxia).

We initially assessed the effects of short-term and continuous hyperoxia (21% O_2_) compared to physioxia (3% O_2_) on cell morphology and total cell number, since oxygen is known to control expansion of NSCs (Braunschweig et al., 2015). NSCs were counted via Countess II with trypan blue staining and additionally via microscopy (Hoechst^+^) after cultivation in physioxia (7 days in 3% pO_2_), continuous hyperoxia (7 days in 21% pO_2_) and short-term hyperoxia (5 days in 3% pO_2_ followed by 2 days in 21% pO_2_). The experiments are based on a previous study from Braunschweig and co-authors, which showed that in NSC cultures grown for 7 days in physioxia (3% pO_2_) and hyperoxia (21% pO_2_) are virtually pure NSC cultures with more than 90% of the cells are Nestin^+^ cells with NSC characteristics and there are no significant differences between the various oxygen tensions (Braunschweig et al., 2015). Visual inspection revealed no morphological changes of NSCs under any oxygen condition, however, clearly showed a reduction of NSCs grown under continuous hyperoxia (Figure 1A). Quantification revealed significantly less NSCs in continuous hyperoxia, but no changes after short-term hyperoxia (Figure 1B; Supplementary Figure S1A). We additionally assessed the total NSC numbers after 5 days of cultivation, which demonstrated a reduction of NSCs in continuous hyperoxia (Supplementary Figure S1B). Taken together, continuous hyperoxia, but not short-term hyperoxia provoked a severely reduced expansion of fetal cortical NSCs.

### 3.2 Continuous and short-term hyperoxia did not change cell death in NSCs

It has been shown that physiological oxygen conditions around 3% pO_2_ are optimal for NSCs survival (Santilli et al., 2010; Braunschweig et al., 2015). Therefore, we analyzed cell death by immunocytochemistry (cleaved caspase 3; CC3) and by flow cytometry (Sub-G0/G1 peak). Under all oxygen conditions, CC3^+^ NSCs were visually scarcely detectable (Figure 1A). The analysis confirmed a generally low percentage of CC3^+^ NSCs (<1% of all cells; Figure 1B). Despite showing a slightly higher proportion of CC3^+^ NSCs under continuous hyperoxia (0.5% ± 0.2%) and short-term hyperoxia (0.5% ± 0.1%) when compared to physioxia (0.3% ± 0.1%), we could not detect any statistical significant differences (p = 0.493, F-value = 1.4, Kruskal–Wallis test; Figure 1B). The analysis of NSCs in the Sub-G0/G1 peak revealed no differences by continuous hyperoxia compared to physioxia (p = 0.096, F-value = 4.7, Kruskal–Wallis test; Figure 1C).

In conclusion, NSCs survived at least as well, if not even better, in physioxia as when cultured under continuous hyperoxia or short-term hyperoxia, however, the proportion of dead NSCs is generally low and does not provide a direct explanation for the significantly reduced cell numbers under continuous hyperoxia.

### 3.3 Hyperoxia differentially affect NSCs proliferation characteristics

Due to the differential effects of continuous vs short-term hyperoxia on NSCs expansion, we analyzed the respective proliferation characteristics in more detail. We thus investigated the proportion of mitotic (phH3^+^) NSCs (Figures 1A, B). The quantification revealed that short-term hyperoxia increased the number of phH3^+^ NSCs, while no changes could be observed under continuous hyperoxia. Subsequently, we analyzed the proportion of NSCs within their respective cell cycle phase and observed major changes under continuous and short-term hyperoxia as compared to physioxia (Figure 1D). Both hyperoxia conditions showed a significant shift of NSCs from G0/G1-phase towards S-phase and G2/M-phase which was more prominent under continuous hyperoxia.

We further conducted gene expression analyses to assess the impact of specific cell cycle genes (Figure 1E). The results showed no significant changes in the relative gene expression of Cyclin D1 (*Ccnd1*), but revealed a significant reduction in Cyclin D2 (*Ccnd2*) expression under both short-term (0.6-fold, p = 0.001) and continuous hyperoxia (0.6-fold, p ≤ 0.001) compared to physioxia. *Ccnd2* is able to activate Cyclin-Dependent Kinase 4 (*Cdk4*) (Pirozzi et al., 2021), which displayed a similarly significant reduction in gene expression under short-term hyperoxia (0.6-fold, p ≤ 0.001) and continuous hyperoxia (0.6-fold, p ≤ 0.001). However, Cyclin-Dependent Kinase 6 (*Cdk6*) showed a significant upregulation in gene expression solely under continuous hyperoxia.

(1.8-fold, p = 0.014) compared to physioxia. To further investigate the G1-to-S-phase transition, we analyzed Cyclin E1 (*Ccne1*), which enters into a complex with Cyclin-Dependent Kinase 2 (*Cdk2*) and controls cell cycle progression and DNA replication through phosphorylation of specific substrates, like several replication factors (Fagundes and Teixeira, 2021). The data indicated a significant increase in *Ccne1* expression under continuous hyperoxia (3.4-fold, p ≤ 0.001) and short-term hyperoxia (1.8-fold, p = 0.042). The associated *Cdk2* demonstrated a significant upregulation in gene expression solely under continuous hyperoxia compared with physioxia (2.4-fold, p = 0.002). Lastly, analysis of the Cyclin-Dependent Kinase Inhibitor 1 (*p21Cip1*) showed a significantly elevated gene expression in response to continuous hyperoxia (2.4-fold, p ≤ 0.001) when compared to physioxia, while short-term hyperoxia showed no effect.

In summary, culturing NSCs under hyperoxia affects their proliferation regarding mitotic activity and cell cycle distribution. Despite severe differences regarding the total number of cells, cell cycle phase distribution and expression of some cell cycle relevant genes showed similar patterns under continuous and short-term hyperoxia.

### 3.4 Short-term hyperoxia dramatically reduces the cell cycle length in NSCs

**FIGURE 2:**
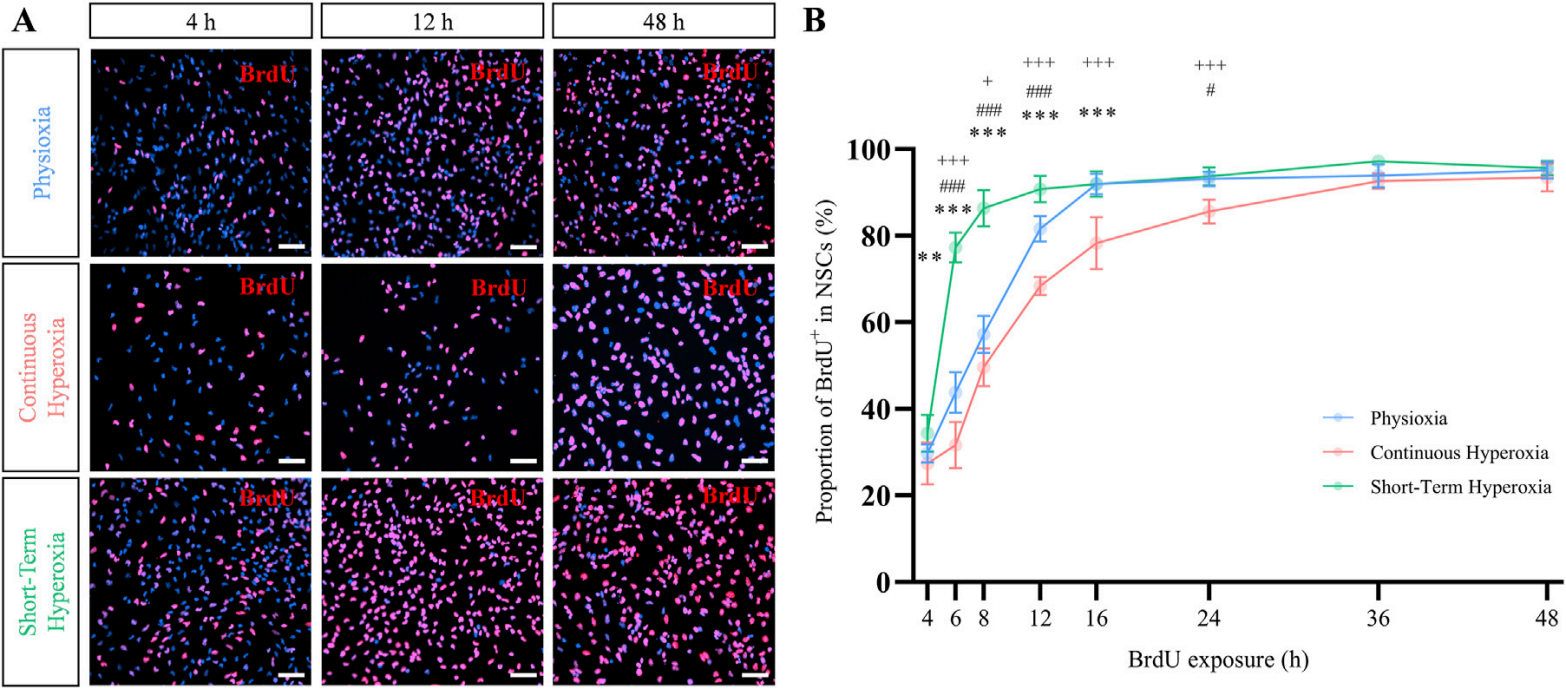
Short-term hyperoxia accelerated BrdU incorporation rate in NSCs. **(A)** Representative fluorescence images of BrdU^+^ (red) NSCs after 4 h (left panel), 12 h (middle panel) and 48 h (right panel) of BrdU exposure time under physioxia (7 days in 3% pO_2_), continuous hyperoxia (7 days in 21% pO_2_) and short-term hyperoxia (5 days in 3% pO_2_ followed by 2 days in 21% pO_2_). Scale bars 50 µm. **(B)** BrdU incorporation rate of NSCs after cultivation in physioxia (blue), continuous hyperoxia (red) or short-term hyperoxia (green). BrdU was added 5 days after seeding and analyzed after 4 h, 6 h, 8 h, 12 h, 16 h, 24 h, 36 h and 48 h. One-way ANOVA with Bonferroni adjusted *post-hoc* tests. * Significance of continuous hyperoxia compared to short-term hyperoxia: *p < 0.05, **p < 0.01, ***p < 0.001; # significance of physioxia compared to short-term hyperoxia: #p < 0.05, ##p < 0.01, ###p < 0.001; + significance of continuous hyperoxia compared to physioxia: +p < 0.05, ++p < 0.01, +++p < 0.001, independent experiments: n = 7 (physioxia), n = 7 (continuous hyperoxia), n = 6 (short-term hyperoxia).

It has been known that NSCs change their cell cycle duration during development (Calegari et al., 2005). Due to differential effects of continuous and short-term hyperoxia regarding the number of NSCs, but only slight changes regarding their proportion within specific cell cycle phases, we hypothesized changes of cell cycle durations and conducted a cumulative BrdU assay to estimated cell cycle phase lengths according to Nowakowski and colleagues (Nowakowski et al., 1989). Representative fluorescence images indicated an increased number of BrdU^+^ cells within short-term hyperoxia treated NSCs which became clearly detectable after 12 h compared to physioxia (Figure 2A). Vice versa, less BrdU^+^ cells were recognizable under continuous hyperoxia. Quantification of BrdU^+^ cells over time confirmed that short-term hyperoxia dramatically enhanced BrdU incorporation rate compared to physioxia and continuous hyperoxia, while physioxia showed a slightly higher BrdU incorporation rate than continuous hyperoxia (Figure 2B). BrdU incorporation reached their plateau after 12 h for short-term hyperoxia, 16 h for physioxia and 36 h for continuous hyperoxia, meaning that at these time points any of the proliferating NSCs have reached the S-phase at least once. The cell cycle phase durations as calculated from the cumulative BrdU incorporation assay are summarized in Table 1.

**TABLE 1:** Cell cycle parameters were obtained by analyzing the rate of BrdU incorporation. The total cell cycle length (T_C_), S-phase duration (T_S_), and the duration of cell cycle phases G0/G1 and G2/M (T_C_–T_S_) are provided in hours. The ratio between the S-phase and total cell cycle length (T_S_/T_C_) and the growth fraction (GF) are expressed as relative values. One-way ANOVA with Bonferroni adjusted *post-hoc t*-tests. Independent experiments: n = 7 (physioxia), n = 7 (continuous hyperoxia), n = 6 (short-term hyperoxia).

	T_C_ (h)	T_S_ (h)	T_C_ - T_S_ (h)	T_S_/T_C_	GF
Physioxia	15.81 ± 0.95	5.01 ± 0.40	10.8 ± 0.56	0.31 ± 0.02	0.94 ± 0.50
Continuous Hyperoxia	26.00 ± 3.07	8.22 ± 1.46	17.79 ± 1.61	0.30 ± 0.01	0.92 ± 0.52
Short-Term Hyperoxia	7.08 ± 0.37	2.80 ± 0.25	4.27 ± 0.12	0.39 ± 0.01	0.94 ± 0.54
One-way ANOVA	p < 0.001, F-value = 22.7	p = 0.003, F-value = 8.4	p < 0.001, F-value = 40.9	p = 0.002, F-value = 8.9	p = 0.051, F-value = 3.6
Bonferroni adjusted *post-hoc* Student’s t-test					
Physioxia vs Continuous Hyperoxia	p = 0.005	p = 0.070	p = 0.012	p = 1.000	p = 0.094
Physioxia vs Short-Term Hyperoxia	p = 0.02	p = 0.350	p < 0.001	p = 0.011	p = 1.000
Continuous Hyperoxia vs Short-Term Hyperoxia	p < 0.001	p = 0.003	p < 0.001	p = 0.003	p = 0.082

NSCs expanding in short-term hyperoxia displayed a significantly shorter cell cycle duration compared to NSCs in physioxia and continuous hyperoxia (p ≤ 0.001, F-value = 22.7, one-way ANOVA; Table 1). This pattern was mirrored regarding S-phase duration, where NSCs in short-term hyperoxia showed the shortest duration compared to physioxia and continuous hyperoxia (p = 0.003, F-value = 8.4, one-way ANOVA). Accordingly, the durations of G1 and G2/M estimated by subtracting the S-phase from the total cell cycle length behaved similarly (p ≤ 0.001, F-value = 40.9, one-way ANOVA; Table 1). However, the proportion of S-phase duration from total cell cycle length (T_S_/T_C_) was significantly longer in short-term hyperoxia cultivated NSCs compared to the other two oxygen conditions (p = 0.002, F-value = 8.9, one-way ANOVA). Importantly, the growth fractions (GFs), which represents the proportion of proliferating cells in a population, were similar under all conditions (p = 0.051, F-value = 3.6, one-way ANOVA). Overall, our data strongly suggest that duration-dependent effects of hyperoxia are regulated via the duration of cell cycle lengths.

## 4 Discussion

Investigations on NSC properties are routinely performed under atmospheric oxygen conditions of 21% pO_2_. However, NSCs physiologically reside in the neurogenic niche at much lower oxygen levels of approx. 2%–5% which potentially limits the interpretation of *in vitro* experiments. To define these time-dependent oxygen effects in more details, we characterized the effects of continuous and short-term hyperoxia (21% pO_2_) as compared to continuous physioxia (3% pO_2_) on cell death and proliferation and on cell cycle kinetics. In general, the oxygen exposure showed strong time-dependent effects on NSC proliferation: When cultured in their physiological condition of 3% pO_2_, NSCs expansion is strongly enhanced (∼2.5-fold increased NSC counts after 7 days of expansion) compared to continuous hyperoxic standard cell culture condition of 21% pO_2_, which is in line with previous observations (Studer et al., 2000; Rodrigues et al., 2010; Braunschweig et al., 2015). Contrary, short-term hyperoxia for 48 h did not affect the number of NSCs when compared to physioxia. This is in close agreement with *in vivo* data on fetal corticogenesis, showing no relevant changes in cortical NSC expansion after short-term hyperoxia and supports gene expression differences to continuous hyperoxia (Wagenführ et al., 2015; Braunschweig et al., 2022; Markert and Storch, 2022).

First we investigated whether NSC expansion is affected by cellular death and analyzed apoptosis via immunohistochemically staining of cleaved caspase 3 (CC3) as well as general cell death via Sub-G0/G1 peak analysis during PI flow cytometry. Here, we could not detect any significant influence of the different oxygen tensions on cell death. This is consistent with previous data on NSCs cultivated as neurospheres in physioxia or hyperoxia for 4 weeks showing a critical role of oxygen via of HIF-1α for expansion/survival of mesencephalic but not cortical NSCs (Milosevic et al., 2005; Milosevic et al., 2007). Indeed, other colleagues also demonstrate no relevant differences of cell death under lower oxygen conditions around 3% pO_2_ when compared to 20% pO_2_ (Santilli et al., 2010; Chen et al., 2017). However, our previous data suggest an increase of NSC death under hyperoxia as compared to physioxia (Braunschweig et al., 2015). The major difference to our present study was a much higher general cell death rate of approx. 9% under physioxia and 14% under hyperoxia but only <1% CC3^+^ apoptotic cells or approx. 2% dead cells within Sub-G0/G1 peak analysis in the present study. The most likely reason for these discrepancies is the change from PLO/Fibronectin coating to PEI/Laminin coating for cell culture surfaces. The fact of extremely low cell death rate in our NSC cultures with no differences between the oxygen conditions in conjunction with the improbable effects on differentiation rate after such a short time *in vitro* are unlikely (Ang, 2006; Braunschweig et al., 2015; Braunschweig et al., 2022), our data strongly suggests that oxygen affects NSCs expansion mainly via changes in their proliferation characteristics.

We thus performed subsequent analyses of mitotic activity using the well-established phH3 marker as well as analysis of cell cycle distribution via flow cytometry in NSCs with respect to oxygen conditions. Corresponding to previous *in vivo* data, short-term hyperoxia led to an increase of mitosis in NSCs, which was also detectable in their cell cycle distribution where more cells could be found within the G2/M-phase as well as S-phase (Wagenführ et al., 2015; Markert and Storch, 2022). Surprisingly, continuous hyperoxia also forced a shift of NSCs from G0/G1-phase to G2/M-phase and S-phase. While this observation is conclusive with mitosis data for short-term hyperoxia, such cell cycle entry was totally unexpected due to the unchanged mitosis data and clearly reduced cell counts in continuous hyperoxia. A reason for this could be found in differences of cell cycle length, which is a key factor for the switch from expansion to differentiation mode of NSCs during brain development (Calegari et al., 2005; Zong et al., 2022). Consistently, the cumulative BrdU assay revealed that continuous hyperoxia significantly increased cell cycle length when compared to physioxia, which explains the increase of S- and G2/M-phase proportion of NSCs. Our estimated cell cycle lengths for physioxia (15.8 h) and continuous hyperoxia (26.0 h) intriguingly reflect the switch from apical progenitors (∼14 h) to basal progenitors (∼23 h) as observed *in vivo* (Arai et al., 2011). However, we could not replicate the *in vivo* specific S-phase lengthening.

Contrary to continuous hyperoxia, short-term hyperoxia has more than halved the duration of the cell cycle (7.1 h) compared to physioxia (15.8 h). To our best knowledge, such short cell cycle durations are currently not known to occur physiologically (Calegari et al., 2005; Arai et al., 2011; Lian et al., 2012; Dee et al., 2016; Fousse et al., 2019; Wang et al., 2021; Molina et al., 2022), but might indicate the relevance of cell cycle kinetics for our previous observations *in vivo* (Wagenführ et al., 2015; Markert and Storch, 2022). Interestingly, the very fast cell cycle of NSCs under short-term hyperoxia went along with an proportional increase of S-phase length which is potentially needed for quality control of DNA replication (Arai et al., 2011). Contributing, it was demonstrated, that S-phase changes and also changes regarding G0-or G2-phase lengthening can affect fate determination of apical progenitors demonstrating the importance of cell cycle length (Arai et al., 2011; Roussat et al., 2023).

Since cell cycle regulation in NSCs seems to be a key player regarding oxygen-dependent regulation of cell expansion, we further analyzed the expression of cell cycle relevant genes, including the master regulator *Cdk2* and the cell cycle inhibitor *p21* (Muller et al., 2023). NSCs grown under continuous hyperoxia showed increased levels of *Cdk2*, but decreased levels of *Cdk4*. Our data thus supports the idea that both *Cdk*-subtypes have redundant functions and are able to compensate for each other as demonstrated in knockout studies of *Cdk2* (Jablonska et al., 2007; Lim and Kaldis, 2012). Additionally, the increased *Cdk2* gene expression after hyperoxia supports our flow cytometry data, since it is known to force cells into the cell cycle, to promote G1/S-phase transition when bound to *Ccne1* and to be highly expressed during S-phase (Dobashi et al., 2000; Stead et al., 2002; Spencer et al., 2013; Urbach and Witte, 2019; Cornwell et al., 2023). Interestingly, *Cdk2* activity is known to be ROS dependent, which might explain its increased expression under continuous hyperoxia (Kirova et al., 2022). Besides *Cdk2*, *Cdk4* and *6* are known to be crucial for cell cycle regulation and both are described as redundant proteins (Ferguson et al., 2000). While *Cdk4* expression is downregulated under both hyperoxia conditions, which is possibly related to a compensation of increased *Cdk2* gene expression, *Cdk6* is highly expressed solely in continuous hyperoxia.

The CDK-inhibitor gene *p21* showed an increased expression under continuous hyperoxia. In general, *p21* together with *Cdk2* are involved in the quiescent decision, where low *p21*/high *Cdk2* forces cells into the cell cycle and high *p21*/low *Cdk2* forces cells into quiescent state even under EGF supplementation (Overton et al., 2014). Besides its direct function in the cell cycle regulation, *p21* additionally binds and downregulates the pluripotency gene *Sox2*, thus contributing to enhanced differentiation of NSCs under continuous hyperoxia as shown by Braunschweig and colleagues (Marques-Torrejon et al., 2013; Braunschweig et al., 2015; Zhu et al., 2021). Contributing, downregulation of Cyclin D2 (*Ccnd2*) together with downregulation of *Cdk4* – as observed under hyperoxia conditions in our experiments –, has been shown to occur physiologically during *in vivo* differentiation of cortical NSCs (Ahn et al., 2004; Niu et al., 2011; Tsunekawa et al., 2012). Overall, our data suggest that elevated oxygen levels clearly affect cell cycle regulation of NSCs. Thereby, NSCs in continuous hyperoxia and, to a lesser extent, in short-term hyperoxia show many regulatory genes which indicate a switch from proliferation towards differentiation. However, our data clearly show that much more effort is needed to decipher cell culture specific regulation of cell cycle-relevant genes which are potentially affected by oxygen, even after short periods of hyperoxygenation.

In conclusion, oxygen − dependent on its exposure duration − affects NSC expansion by regulating cell cycle length. Of note, already an exposure to atmospheric oxygen levels for a few hours dramatically shifts NSCs from G0/G1-phase into S-phase and G2/M-phase. The exact molecular pathways mediating these oxygen effects need to be elucidated in future studies. Together, our findings further demonstrate the beneficial effects of continuous physioxia for NSC expansion and further stress the importance of adequate oxygen conditions similar to those found within the neurogenic niche for studying cell biology properties of NSCs, such as cell cycle regulation, in *in vitro* experiments.

## Data Availability

The raw data supporting the conclusions of this article will be made available by the authors, without undue reservation.

## Supplementary material

SUPPLEMENTARY FIGURE S1 Further analysis of cell counts using immunohistochemistry showed a reduction of NSCs growth under continuous hyperoxia.

## References

[B1] Ahn J. I. Lee K. H. Shin D. M. Shim J. W. Kim C. M. Kim H. (2004). Temporal expression changes during differentiation of neural stem cells derived from mouse embryonic stem cell. J. Cell Biochem. 93 (3), 563–578. 10.1002/jcb.20168 15378605

[B2] Ang S. L. (2006). Transcriptional control of midbrain dopaminergic neuron development. Development 133 (18), 3499–3506. 10.1242/dev.02501 16899537

[B3] Arai Y. Pulvers J. N. Haffner C. Schilling B. Nusslein I. Calegari F. (2011). Neural stem and progenitor cells shorten S-phase on commitment to neuron production. Nat. Commun. 2, 154. 10.1038/ncomms1155 21224845 PMC3105305

[B4] Braunschweig L. Lanto J. Meyer A. K. Markert F. Storch A. (2022). Serial gene expression profiling of neural stem cells shows transcriptome switch by long-term physioxia from metabolic adaption to cell signaling profile. Stem Cells Int. 2022, 6718640. 10.1155/2022/6718640 36411871 PMC9675612

[B5] Braunschweig L. Meyer A. K. Wagenführ L. Storch A. (2015). Oxygen regulates proliferation of neural stem cells through Wnt/β-catenin signalling. Mol. Cell. Neurosci. 67, 84–92. 10.1016/j.mcn.2015.06.006 26079803

[B6] Calegari F. Haubensak W. Haffner C. Huttner W. B. (2005). Selective lengthening of the cell cycle in the neurogenic subpopulation of neural progenitor cells during mouse brain development. J. Neurosci. 25 (28), 6533–6538. 10.1523/jneurosci.0778-05.2005 16014714 PMC6725437

[B7] Chen C. C. Hsia C. W. Ho C. W. Liang C. M. Chen C. M. Huang K. L. (2017). Hypoxia and hyperoxia differentially control proliferation of rat neural crest stem cells via distinct regulatory pathways of the HIF1α-CXCR4 and TP53-TPM1 proteins. Dev. Dyn. 246 (3), 162–185. 10.1002/dvdy.24481 28002632

[B8] Chen H. L. Pistollato F. Hoeppner D. J. Ni H. T. McKay R. D. Panchision D. M. (2007). Oxygen tension regulates survival and fate of mouse central nervous system precursors at multiple levels. Stem Cells 25 (9), 2291–2301. 10.1634/stemcells.2006-0609 17556599

[B9] Chou F. S. Chen C. Y. Lee A. C. Wang P. S. (2022). Impaired cell cycle progression and self-renewal of fetal neural stem and progenitor cells in a murine model of intrauterine growth restriction. Front. Cell Dev. Biol. 10, 821848. 10.3389/fcell.2022.821848 35903551 PMC9314876

[B10] Cornwell J. A. Crncec A. Afifi M. M. Tang K. Amin R. Cappell S. D. (2023). Loss of CDK4/6 activity in S/G2 phase leads to cell cycle reversal. Nature 619 (7969), 363–370. 10.1038/s41586-023-06274-3 37407814 PMC10338338

[B11] Dee A. Li K. Heng X. Guo Q. Li J. Y. (2016). Regulation of self-renewing neural progenitors by FGF/ERK signaling controls formation of the inferior colliculus. Development 143 (20), 3661–3673. 10.1242/dev.138537 27578777 PMC5087642

[B12] Dey D. Shrivastava V. Joshi D. Singal C. M. S. Tyagi S. Bhat M. A. (2023). Hypoxia induces early neurogenesis in human fetal neural stem cells by activating the WNT pathway. Mol. Neurobiol. 60 (5), 2910–2921. 10.1007/s12035-023-03248-4 36749560

[B13] Dings J. Meixensberger J. Jager A. Roosen K. (1998). Clinical experience with 118 brain tissue oxygen partial pressure catheter probes. Neurosurgery 43 (5), 1082–1095. 10.1097/00006123-199811000-00045 9802852

[B14] Dobashi Y. Shoji M. Kitagawa M. Noguchi T. Kameya T. (2000). Simultaneous suppression of cdc2 and cdk2 activities induces neuronal differentiation of PC12 cells. J. Biol. Chem. 275 (17), 12572–12580. 10.1074/jbc.275.17.12572 10777547

[B15] Erecinska M. Silver I. A. (2001). Tissue oxygen tension and brain sensitivity to hypoxia. Respir. Physiol. 128 (3), 263–276. 10.1016/s0034-5687(01)00306-1 11718758

[B16] Fagundes R. Teixeira L. K. (2021). Cyclin E/CDK2: DNA replication, replication stress and genomic instability. Front. Cell Dev. Biol. 9, 774845. 10.3389/fcell.2021.774845 34901021 PMC8652076

[B17] Ferguson K. L. Callaghan S. M. O'Hare M. J. Park D. S. Slack R. S. (2000). The Rb-CDK4/6 signaling pathway is critical in neural precursor cell cycle regulation. J. Biol. Chem. 275 (43), 33593–33600. 10.1074/jbc.M004879200 10915795

[B18] Forristal C. E. Wright K. L. Hanley N. A. Oreffo R. O. C. Houghton F. D. (2010). Hypoxia inducible factors regulate pluripotency and proliferation in human embryonic stem cells cultured at reduced oxygen tensions. Reprod. Camb. Engl. 139 (1), 85–97. 10.1530/rep-09-0300 PMC2791494 19755485

[B19] Fousse J. Gautier E. Patti D. Dehay C. (2019). Developmental changes in interkinetic nuclear migration dynamics with respect to cell-cycle progression in the mouse cerebral cortex ventricular zone. J. Comp. Neurol. 527 (10), 1545–1557. 10.1002/cne.24641 30682231

[B20] Francis K. R. Wei L. (2010). Human embryonic stem cell neural differentiation and enhanced cell survival promoted by hypoxic preconditioning. Cell Death Dis. 1 (2), e22. 10.1038/cddis.2009.22 21364630 PMC3032335

[B21] Jablonska B. Aguirre A. Vandenbosch R. Belachew S. Berthet C. Kaldis P. (2007). Cdk2 is critical for proliferation and self-renewal of neural progenitor cells in the adult subventricular zone. J. Cell Biol. 179 (6), 1231–1245. 10.1083/jcb.200702031 18086919 PMC2140044

[B22] Kirova D. G. Judasova K. Vorhauser J. Zerjatke T. Leung J. K. Glauche I. (2022). A ROS-dependent mechanism promotes CDK2 phosphorylation to drive progression through S phase. Dev. Cell 57 (14), 1712–1727.e9. 10.1016/j.devcel.2022.06.008 35809563 PMC9616724

[B23] Lange C. Turrero Garcia M. Decimo I. Bifari F. Eelen G. Quaegebeur A. (2016). Relief of hypoxia by angiogenesis promotes neural stem cell differentiation by targeting glycolysis. EMBO J. 35 (9), 924–941. 10.15252/embj.201592372 26856890 PMC5207321

[B24] Lian G. Lu J. Hu J. Zhang J. Cross S. H. Ferland R. J. (2012). Filamin a regulates neural progenitor proliferation and cortical size through Wee1-dependent Cdk1 phosphorylation. J. Neurosci. 32 (22), 7672–7684. 10.1523/jneurosci.0894-12.2012 22649246 PMC3368379

[B25] Lim S. Kaldis P. (2012). Loss of Cdk2 and Cdk4 induces a switch from proliferation to differentiation in neural stem cells. Stem Cells 30 (7), 1509–1520. 10.1002/stem.1114 22532528

[B26] Markert F. Müller L. Badstübner-Meeske K. Storch A. (2020). Early chronic intermittent maternal hyperoxygenation impairs cortical development by inhibition of pax6-positive apical progenitor cell proliferation. J. Neuropathol. Exp. Neurol. 79 (11), 1223–1232. 10.1093/jnen/nlaa072 32929481

[B27] Markert F. Storch A. (2022). Hyperoxygenation during mid-neurogenesis accelerates cortical development in the fetal mouse brain. Front. Cell Dev. Biol. 10, 732682. 10.3389/fcell.2022.732682 35372333 PMC8969024

[B28] Marques-Torrejon M. A. Porlan E. Banito A. Gomez-Ibarlucea E. Lopez-Contreras A. J. Fernandez-Capetillo O. (2013). Cyclin-dependent kinase inhibitor p21 controls adult neural stem cell expansion by regulating Sox2 gene expression. Cell Stem Cell 12 (1), 88–100. 10.1016/j.stem.2012.12.001 23260487 PMC3714747

[B29] Milosevic J. Maisel M. Wegner F. Leuchtenberger J. Wenger R. H. Gerlach M. (2007). Lack of hypoxia-inducible factor-1 alpha impairs midbrain neural precursor cells involving vascular endothelial growth factor signaling. J. Neurosci. 27 (2), 412–421. 10.1523/jneurosci.2482-06.2007 17215402 PMC6672078

[B30] Milosevic J. Schwarz S. C. Krohn K. Poppe M. Storch A. Schwarz J. (2005). Low atmospheric oxygen avoids maturation, senescence and cell death of murine mesencephalic neural precursors. J. Neurochem. 92 (4), 718–729. 10.1111/j.1471-4159.2004.02893.x 15686473

[B31] Molina A. Bonnet F. Pignolet J. Lobjois V. Bel-Vialar S. Gautrais J. (2022). Single-cell imaging of the cell cycle reveals CDC25B-induced heterogeneity of G1 phase length in neural progenitor cells. Development 149 (11), dev199660. 10.1242/dev.199660 35588250

[B32] Muller L. Gutschner T. Hatzfeld M. (2023). Going only half the way: cell cycle exit after the G1 restriction point. Signal Transduct. Target Ther. 8 (1), 440. 10.1038/s41392-023-01692-1 38040673 PMC10692125

[B33] Niu W. Zou Y. Shen C. Zhang C. L. (2011). Activation of postnatal neural stem cells requires nuclear receptor TLX. J. Neurosci. 31 (39), 13816–13828. 10.1523/JNEUROSCI.1038-11.2011 21957244 PMC3192402

[B34] Nowakowski R. S. Lewin S. B. Miller M. W. (1989). Bromodeoxyuridine immunohistochemical determination of the lengths of the cell cycle and the DNA-synthetic phase for an anatomically defined population. J. Neurocytol. 18 (3), 311–318. 10.1007/BF01190834 2746304

[B35] Overton K. W. Spencer S. L. Noderer W. L. Meyer T. Wang C. L. (2014). Basal p21 controls population heterogeneity in cycling and quiescent cell cycle states. Proc. Natl. Acad. Sci. U. S. A. 111 (41), E4386–E4393. 10.1073/pnas.1409797111 25267623 PMC4205626

[B36] Pirozzi F. Lee B. Horsley N. Burkardt D. D. Dobyns W. B. Graham J. M. Jr. (2021). Proximal variants in CCND2 associated with microcephaly, short stature, and developmental delay: a case series and review of inverse brain growth phenotypes. Am. J. Med. Genet. A 185 (9), 2719–2738. 10.1002/ajmg.a.62362 34087052 PMC8725575

[B37] Rodrigues C. A. Diogo M. M. da Silva C. L. Cabral J. M. (2010). Hypoxia enhances proliferation of mouse embryonic stem cell-derived neural stem cells. Biotechnol. Bioeng. 106 (2), 260–270. 10.1002/bit.22648 20014442

[B38] Roussat M. Jungas T. Audouard C. Omerani S. Medevielle F. Agius E. (2023). Control of G(2) phase duration by CDC25B modulates the switch from direct to indirect neurogenesis in the neocortex. J. Neurosci. 43 (7), 1154–1165. 10.1523/jneurosci.0825-22.2022 36596698 PMC9962783

[B39] Santilli G. Lamorte G. Carlessi L. Ferrari D. Rota Nodari L. Binda E. (2010). Mild hypoxia enhances proliferation and multipotency of human neural stem cells. PLoS One 5 (1), e8575. 10.1371/journal.pone.0008575 20052410 PMC2797394

[B40] Schneider C. Krischke G. Rascher W. Gassmann M. Trollmann R. (2012). Systemic hypoxia differentially affects neurogenesis during early mouse brain maturation. Brain Dev. 34 (4), 261–273. 10.1016/j.braindev.2011.07.006 21824737

[B41] Spencer S. L. Cappell S. D. Tsai F. C. Overton K. W. Wang C. L. Meyer T. (2013). The proliferation-quiescence decision is controlled by a bifurcation in CDK2 activity at mitotic exit. Cell 155 (2), 369–383. 10.1016/j.cell.2013.08.062 24075009 PMC4001917

[B42] Stead E. White J. Faast R. Conn S. Goldstone S. Rathjen J. (2002). Pluripotent cell division cycles are driven by ectopic Cdk2, cyclin A/E and E2F activities. Oncogene 21 (54), 8320–8333. 10.1038/sj.onc.1206015 12447695

[B43] Studer L. Csete M. Lee S.-H. Kabbani N. Walikonis J. Wold B. (2000). Enhanced proliferation, survival, and dopaminergic differentiation of CNS precursors in lowered oxygen. J. Neurosci. 20 (19), 7377–7383. 10.1523/jneurosci.20-19-07377.2000 11007896 PMC6772777

[B44] Tsunekawa Y. Britto J. M. Takahashi M. Polleux F. Tan S. S. Osumi N. (2012). Cyclin D2 in the basal process of neural progenitors is linked to non-equivalent cell fates. EMBO J. 31 (8), 1879–1892. 10.1038/emboj.2012.43 22395070 PMC3343330

[B45] Urbach A. Witte O. W. (2019). Divide or commit - revisiting the role of cell cycle regulators in adult hippocampal neurogenesis. Front. Cell Dev. Biol. 7, 55. 10.3389/fcell.2019.00055 31069222 PMC6491688

[B46] Wagenführ L. Meyer A. K. Braunschweig L. Marrone L. Storch A. (2015). Brain oxygen tension controls the expansion of outer subventricular zone-like basal progenitors in the developing mouse brain. Dev. Camb. Engl. 142 (17), 2904–2915. 10.1242/dev.121939 26329599

[B47] Wagenfuhr L. Meyer A. K. Marrone L. Storch A. (2016). Oxygen tension within the neurogenic niche regulates dopaminergic neurogenesis in the developing midbrain. Stem Cells Dev. 25 (3), 227–238. 10.1089/scd.2015.0214 26577812 PMC4742976

[B48] Wang A. Wang J. Tian K. Huo D. Ye H. Li S. (2021). An epigenetic circuit controls neurogenic programs during neocortex development. Development 148 (22), dev199772. 10.1242/dev.199772 35020876

[B49] Zhu L.-L. Wu L.-Y. Yew D. T. Fan M. (2005). Effects of hypoxia on the proliferation and differentiation of NSCs. Mol. Neurobiol. 31 (1-3), 231–242. 10.1385/mn:31:1-3:231 15953824

[B50] Zhu Q. Chen L. Li Y. Huang M. Shao J. Li S. (2021). Rack1 is essential for corticogenesis by preventing p21-dependent senescence in neural stem cells. Cell Rep. 36 (9), 109639. 10.1016/j.celrep.2021.109639 34469723

[B51] Zong N. Wang M. Fu Y. Shen D. Yu Y. C. (2022). Cell-cycle length of medial ganglionic eminence progenitors contributes to interneuron fate. Protein Cell 13 (2), 141–147. 10.1007/s13238-021-00851-w 34043145 PMC8783944

