# Supplementary material for: Hyperoxia shows duration-dependent effects on the lengths of cell cycle phases in fetal cortical neural stem cells
Source: Front Cell Dev Biol. 2025 Jan 28;13:1546131. doi: 10.3389/fcell.2025.1546131 (PMC11811091; doi:10.3389/fcell.2025.1546131)
Supplement: Supplementary file 1 [file DataSheet1.docx]

Supplementary Material

**Hyperoxia shows duration-dependent effects on the lengths of cell cycle phases in fetal cortical neural stem cells**

**Jennifer Lanto, Monika Maria Nicole Vehlken, Valeriia Abramenko, Alexander Storch, Franz Markert* Correspondence:** Franz Markert: franz.markert@med.uni-rostock.de

## Supplementary Figures

- **Supplementary Figure S1.:** Analysis of cell counts using immunohistochemistry showed a reduction of NSCs growth under continuous hyperoxia.


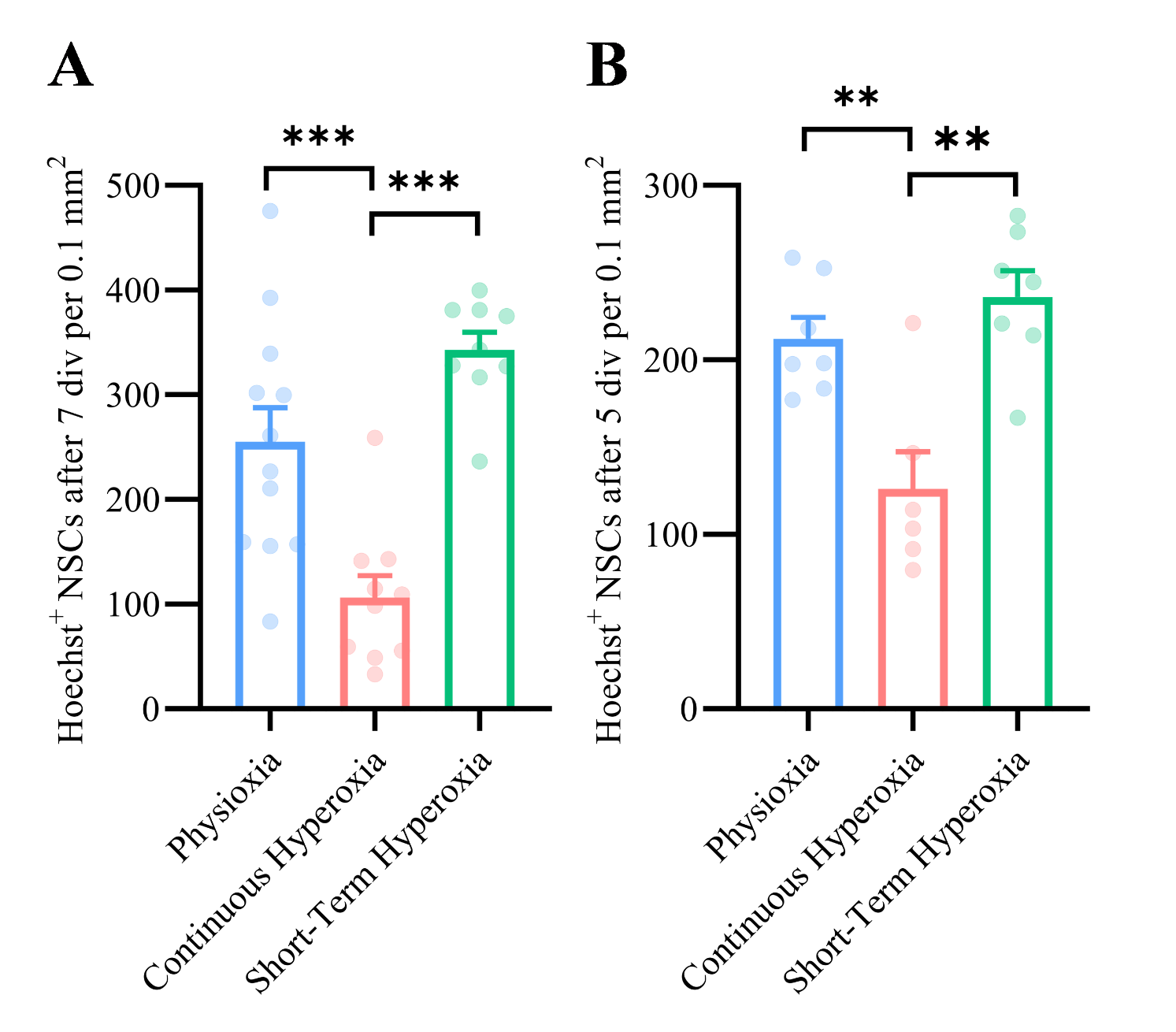


**Supplementary Figure S1.** Analysis of cell counts using immunohistochemistry showed a reduction of NSCs growth under continuous hyperoxia. (**A**) Quantitative analyses of cell counts using immunofluorescence staining via Hoechst^+^ NSCs per 0.1 mm^2^ after **7** days of cultivation and (**B**) after 5 days under different oxygen conditions; physioxia (7 days in 3% pO_2_, blue), continuous hyperoxia (7 days in 21% pO_2_, red), and short-term hyperoxia (5 days in 3% pO_2_ followed by 2 days in 21% pO_2_, green). One-way ANOVA with Bonferroni adjusted *post-hoc* tests. ***p < 0.001, **p < 0.01, independent experiments: n ≥ 7 (physioxia), n ≥ 7 (continuous hyperoxia), n ≥ 7 (short-term hyperoxia).
